# Cervical and shoulder postural assessment of adolescents between 15 and 17 years old and association with upper quadrant pain

**DOI:** 10.1590/bjpt-rbf.2014.0027

**Published:** 2014

**Authors:** Rodrigo M. Ruivo, Pedro Pezarat-Correia, Ana I. Carita

**Affiliations:** 1 Centro Interdisciplinar de Performance Humana, Faculdade de Motricidade Humana, Universidade de Lisboa, Lisbon, Portugal; 2 Secção Autónoma de Métodos Matemáticos, Faculdade de Motricidade Humana, Universidade de Lisboa, Lisbon, Portugal

**Keywords:** adolescents, cervical, photogrammetry, rehabilitation, posture, shoulder

## Abstract

**Background:**

There is sparse literature that provides evidence of cervical and shoulder postural alignment of 15 to 17-year-old adolescents and that analyzes sex differences.

**Objectives:**

To characterize the postural alignment of the head and shoulder in the sagittal plane of 15 to 17-year-old Portuguese adolescents in natural erect standing and explore the relationships between three postural angles and presence of neck and shoulder pain.

**Method:**

This cross-sectional study was conducted in two secondary schools in Portugal. 275 adolescent students (153 females and 122 males) aged 15 to 17 were evaluated. Sagittal head, cervical, and shoulder angles were measured with photogrammetry and PAS software. The American Shoulder and Elbow Surgeons Shoulder Assessment (ASES) was used to assess shoulder pain, whereas neck pain was self-reported with a single question.

**Results:**

Mean values of sagittal head, cervical, and shoulder angles were 17.2±5.7, 47.4±5.2, and 51.4±8.5º, respectively. 68% of the participants revealed protraction of the head, whereas 58% of them had protraction of the shoulder. The boys showed a significantly higher mean cervical angle, and adolescents with neck pain revealed lower mean cervical angle than adolescents without neck pain. 53% of the girls self-reported regular neck pain, contrasting with 19% of the boys.

**Conclusions:**

This data shows that forward head and protracted shoulder are common postural disorders in adolescents, especially in girls. Neck pain is prevalent in adolescents, especially girls, and it is associated with forward head posture.

## Introduction

Posture has been defined as the alignment of the body segments at a particular time^1^ and is an important health indicator^2^. It must correspond to a specific body position in space which minimizes anti-gravity stresses on body tissues^3^. Inadequate posture consists of poor interrelations between parts of the body^4^. These imperfect interrelations cause muscle tension and shortening, which makes appropriate joint movements more difficult to achieve^5^ and may cause pain.

Epidemiological studies have shown a high prevalence of spinal postural deviations in children and adolescents^6^ ^,^ ^7^, with forward head posture (FHP) and protracted shoulder (PS) posture being two of the most common postural deviations^7^. FHP is commonly defined as the protrusion of the head in the sagittal plane so that the head is placed anterior to the trunk^8^. It can occur because of anterior translation of the head, lower cervical flexion or both, and it is claimed to be associated with an increase in upper cervical extension^8^. It is associated with shortening of the upper trapezius, the posterior cervical extensor muscles, the sternocleidomastoid muscle and the levator scapulae muscle^9^. It is thought that adolescents or patients with neck pain (NP) have a more forward head posture, thus a smaller craniovertebral (CV) angle in standing, than age-matched pain-free participants^10^. PS is a forward displacement of the acromion with reference to the 7^th^ cervical spinous process, frequently associated with a protracted, anterior tilted and internally rotated scapula and with a tightness of the pectoralis minor muscle^11^.

To study the misalignments outlined above, the photographic measurement of sagittal postures of cervical spine and shoulder is becoming more widespread, with several studies confirming the high reliability of photogrammetry^2^ ^,^ ^9^ ^,^ ^12^ ^-^ ^14^. To assist with posture assessment from digitized images, specific software has been developed such as PAS/SAPO (Postural Assessment Software)^12^.

Based on the knowledge that the current literature is still sparse in the characterization of the postural alignment of adolescents in a large sample size and that there is no concrete information on the relationship between neck and shoulder pain and sagittal posture of the spine in a standing position, we defined the following objectives for this study: 1) to characterize the postural alignment of the head and shoulders in the sagittal plane of 15 to 17-year-old Portuguese adolescents in natural erect standing; 2) to find the relationship (if any) between the postural angles studied and neck and shoulder pain; and 3) to analyze sex differences in the postural angles and neck and shoulder pain.

The findings of this study may give researchers further information about cervical and shoulder postural alignment of a specific age group and will help to evaluate the relationship between neck and shoulder pain and posture. Moreover, the results may help to improve the management of patients with neck pain. This study has the advantage of having evaluated a far larger sample than other studies^6^ ^,^ ^15^ and analyzed sex differences.

## Method

### Participants

This cross-sectional study was conducted in two public secondary schools, Lumiar Secondary School and Padre Antonio Vieira Secondary School, located in the city of Lisbon, Portugal. Male and female adolescent students between the ages of 15 and 17 years were eligible to participate. The justification of the ages is to avoid the effects of the pubertal growth spurt. Participants were excluded if they had visual deficits, diagnosed balance disorders, musculoskeletal pathologies (e.g. history of shoulder surgery, cervical or thoracic fracture), were non-ambulatory, displayed functional or structural scoliosis, or had excessive thoracic kyphosis. Given these criteria, a total of 275 adolescent students (146 females and 129 males) aged 15, 16, or 17 years old [15.76±1.08 y] from 17 different classes (nine from the 10^th^ grade, seven from the 11^th^ grade, and one from the 12^th^ grade) were evaluated and included in the study.

The participation of all students was voluntary, and written informed consent was obtained from all participants, and their parents or legal guardians. The study was approved by the Research Ethic s Committee of the Faculty of Human Kinetics from Universidade de Lisboa, Lisbon, Portugal (approval number: 5/2013).

### Procedures

### Posture alignment assessment

**Figure 1:**
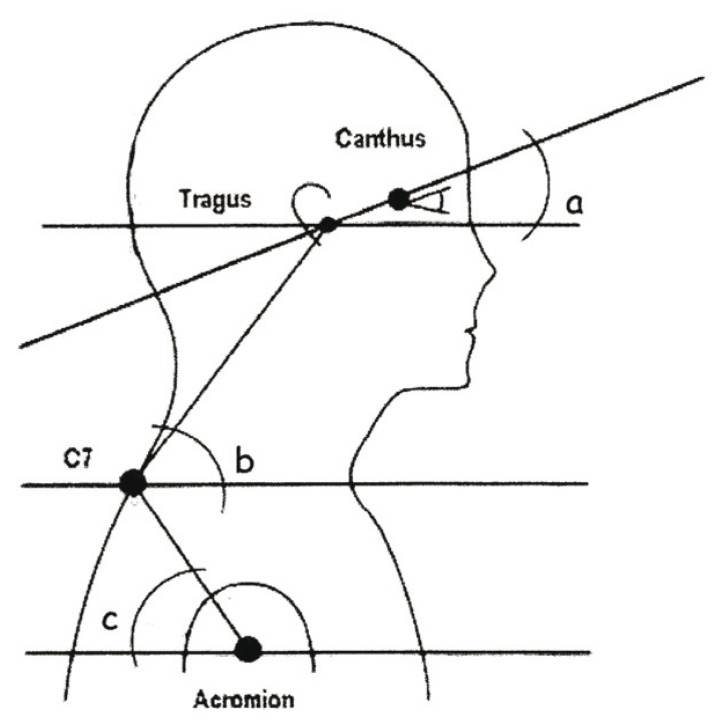
Adhesive marker placement and postural angles. a sagittal head; b cervical angle; c shoulder angle.

Standing cervical and shoulder posture was measured with photogrammetry and PAS software. When compared to radiographs using the LODOX, the photographs provide valid and reliable indicators of the spine^6^. Also the software PAS has proven to be valid and reliable^12^. Three angles of measurement were used - sagittal head angle (HT), cervical angle (CV), and shoulder angle (SH) (Figure 1) - and obtained in the sagittal view as follows:

Sagittal head angle - The angle formed at the intersection of a horizontal line through the tragus of the ear and a line joining the tragus of the ear and the lateral canthus of the eye.

Cervical angle - The cervical angle is highly reliable to assess the forward head position^4^. It is the angle formed at the intersection of a horizontal line through the spinous process of C7 and a line to the tragus of the ear. In this study, if the angle was less than 50º, the participant was considered to have forward head posture. The selection of 50º as a reference angle was guided by the studies of Diab and Moustafa^16^ and Yip et al.^17^, with the latter reporting 55.02±2.86 as a normal range. As is well known, subjects with FHP have a significantly smaller cervical angle when compared with normal subjects^18^.

Shoulder angle - The angle formed at the intersection of the line between the midpoint of the humerus and spinous process of C7 and the horizontal line through the midpoint of the humerus. In the present study, we considered 52º as the reference angle based on Thigpen et al.^19^ who evaluated 310 participants in a standing position and reported 2.6º±15.3 as a normal range, and Brink et al.^20^, who evaluated 15 to 17 year-olds and reported a mean shoulder angle value of 51.35º±17.2º, and based on the premise that subjects with protracted shoulder have a significantly smaller shoulder angle when compared with normal subjects^15^. We considered an individual to have PS if the angle was less than 52º.

All measurements were taken by the same researcher who was experienced in the assessment of postural alignment. The photographing took place in the gymnasium of the 2 secondary schools with the areas arranged identically. Landmarks were placed on the floor to ensure the same positioning of all subjects in front of the camera and to ensure that the subject was aligned perpendicular to the camera. A landmark was placed in front of a white wall to ensure a contrast of the subjects against the background. One Canon Power Shot A4000 IS was supported on a Manfrotto tripod, model 055 CLB, three meters away from the line marking the position of the subject. The height of the tripod was adjusted so the middle of the objective lens was 130 cm above the ground. A calibration board was placed in the field of view and aligned with the subject to allow referencing of horizontal and vertical axes from the photographs. The calibration board also displayed each subject's identification number. For positioning, the adolescent was instructed to stand comfortably in a normal standing position and to look straight ahead. Marks on the floor ensured that all subjects were in the same place.

Before photographing, the researcher put reflective markers (styrofoam balls with 20 mm diameter) on the following anatomical points on the right side of the subject´s body: tragus of the ear, lateral canthus of the eye, spinous process of C7, and midpoint of the humerus. With these markers we were able to calculate the sagittal head angle, cervical angle, and shoulder angle.

To enable precise positioning of the markers we instructed the subjects to wear tight shorts and sleeveless t-shirts and to tie their hair back when needed. The procedure was always performed by the same researcher, who was blinded to the subjects' condition. Each person was asked to look straight ahead and to march on the spot five times before each picture was taken^21^ to capture the participant's natural head-on-trunk and shoulder alignment. Each picture was taken within five seconds of the marching sequence, in a lateral view, with the right side of the subject photographed for the right hand-dominant participants and the left side for the left-hand dominant participants. The dominant arm was defined as the most used in daily activities. The photographic analysis was subsequently performed using PAS, which determined the coordinates of the anatomical points on the photographs. The zoom was standardized at 200% and the angles were measured in degrees. One researcher undertook all scanning and digitizing to eliminate inter-examiner error. The data were submitted to descriptive statistical analysis, and quantitative values for head and upper member angles were obtained. PAS has already been shown to be valid and reliable^12^.

### Self-assessment of shoulder pain and function and neck pain

The American Shoulder and Elbow Surgeons Shoulder Assessment (ASES) form was translated and cross-culturally adapted to the Portuguese language. This Portuguese version was then used to record the presence of shoulder pain and function in the subjects. The questionnaire addressed self-evaluation of pain using a visual analog scale and activities of daily living questionnaire. A high total score indicates low perceived pain and low dysfunction in activities of daily living. After the postural assessment and administration of the ASES questionnaire, the students were asked to answer yes or no to the following question: do you feel neck pain regularly? With this question we also wanted to address neck pain as an outcome measure.

### Reliability study

A separate preparatory study to confirm the inter- and intra-rater reliability of computerized photogrammetry using the PAS was performed. The study sample consisted of 17 subjects from the 10^th^ grade. Three physical therapists (all men from 26 to 32 years old), who had used the PAS/SAPO before but were not regular users, were invited to participate as raters. Each student was photographed in the same conditions as detailed before in the main study, and pictures were taken of the participants in random order. Using the PAS, the three raters took the measurements, which were then used to calculate the inter-rater reliability. These procedures were repeated one week later by therapist A, and the results were compared to assess the intra-rater reliability.

### Statistical analysis

All statistical analyses were performed using specific software (SPSS version 20), and the α value was defined in 0.05. Intra-rater reliability was assessed using type 2.1 intraclass correlation coefficient (ICC), whereas the inter-rater reliability was assessed using ICC(3.1).

The Shapiro-Wilk test was used to assess normality. To analyze differences between sexes and between patients with and without neck pain (NP) in the three postural angles and ASES scores, the independent-samples t-test was applied. A chi-square test was used to assess the relationship between the forward head and cervical pain. Relationships between the three postural angles and ASES were examined by calculating Spearman's rho correlation coefficient (r_s_).

## Results

### Reliability study

The reliability of the photographic measurement is shown in Table 1. A total of 17 subjects (14 females and 3 males) aged 15 to 17 years were recruited for the reliability study. The ICC (2.1) values for the shoulder angle and for the cervical angle reported good reliability, with 0.78 and 0.66 respectively, whereas the values for the HT angle (0.83) revealed very good intra-rater reliability. All the ICC (3.1) values for the three angles, in the inter-rater reliability, reported a very good reliability, with the SEMs of the photographic measurement ranging between 1.64 and 2.35.

**Table 1:** Intra-rater and inter-rater reliability findings: ICC and SEM values for all angles.

Measurement	Intra-rater reliability			Inter-rater reliability		
	ICC (95% CI)	SEM	MDC	ICC (95% CI)	SEM	MDC
Sagitall Head Angle Cervical Angle Shoulder Angle	0.83 (0.60-0.94) 0.66 (0.26-0.87) 0.78 (0.49-0.92)	2.72 3.54 4.03	7.54 9.81 11.18	0.88 (0.75-0.95) 0.87 (0.74-0.95) 0.96 (0.92-0.99)	2.35 1.85 1.64	6.51 5.13 4.55

ICC - Intraclass correlation coefficient

SEM - standard error of measurement

### Experimental study

### Sample

A total of 275 adolescents, 153 girls and 122 boys (age 15±1 year), participated in the study. Sex and descriptive values for the three postural angles and ASES scores are described in Table 2.

**Table 2:** Descriptive values for the postural angles and ASES scores (n=275) and effect of gender and neck pain in postural angles and ASES scores.

	Overall					Females	Males		
	All (n=275)	No NP (n=170)	NP (n=105)	t	p	All (n=153)	All (n=122)	t	p
Sagittal head tilt angle	17.2±5.7	17.6±5.7	16.4±5.7	1.76	0.008	16.15±5.3	18.4±6.03	–3.3	0.001
Cervical angle	47.4±5.174	47.96±4.79	46.46 ±5.6	2.358	0.019 *	46.55±5.2	48.43±4.91	–3.05	0.002*
Shoulder angle	51.4±8.548	50.95 ±8.18	52.24±9.13	–1.219	0.224	51.09±8.27	51.88±8.92	–0.765	0.445
ASES Scores (right)	93.3±9.53	95.06±6.68	90.46±12.40	3.99	0.000*	92.31±10.7	94.55±7.59	3.136	0.053
ASES Scores (left)	91.6± 9.38	93.13±7.75	89.10±11.14	3.52	0.000*	90.46±9.99	93.01±8.37	1.252	0.025*
	Females				Males				
	No NP (n=72)	NP (n=81)	t	p	No NP (n=98)	NP (n=24)	t	p	
Sagittal head tilt angle	16.5±5.1	15.8±5.5	0.67	0.5	18.5±6.0	18.1±6.3	0.3	0.76	
Cervical angle	47.38±4.76	45.8±5.6	1.86	0.0048*	48.38±4.79	48.63±5.5	–0.221	0.825	
Shoulder angle	50.72±7.72	51.4±8.78	–0.52	0.603	51.12±8.4	55.02±9.89	–1.944	0.054	
ASES Scores (right)	94.92±5.85	89.98±13.33	2.91	0.004*	95.16±7.25	92.07±8.57	1.800	0.074*	
ASES Scores (left)	92.53±6.96	88.62±11.8	2.45	0.015*	93.56±8.29	90.76±8.51	1.479	0.142	

ASES - American shoulder and elbow surgeons shoulder assessment

NP - neck pain

* Statistically significant difference (p<0.05).

**Figure 2:**
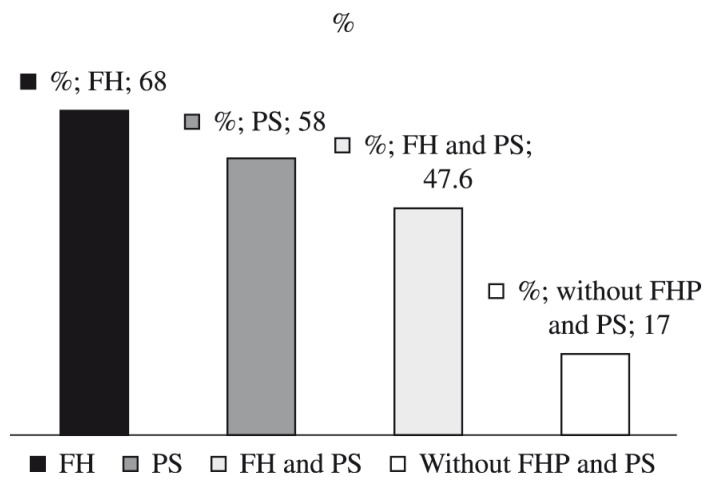
Percentage of students experiencing forward head and/ or protracted shoulder (PS).

Bearing in mind the reference values outlined before, of the 275 adolescents studied, 188 (68%) had forward head (FH) with a cervical angle less than 50º, while 131 (58%) had a shoulder angle less than 52º, revealing a PS. These values are shown in Figure 2.

### Sex, neck pain, postural angles and ASES

The examination of the head and shoulder posture measurements to identify the effect of sex and NP on postural angles and ASES scores using the t-test is reported in Table 2. Significant differences were observed between boys and girls with respect to the HT angle and the CV angle, with the boys reporting a higher mean value (18.4±6.03 vs 16.15±5.31, and 48.43±4.91 vs 46.55±5.24, respectively).

105 adolescents (38.2%) of the 275 reported having NP regularly. The overall NP group showed a significantly lower mean CV angle (46.5±5.6 vs 47.9±4.79), whereas no statistically significant difference was found between patients and pain-free participants for the HT angle (t=1.76, *P*>.05) and SH angle (t=-1.2, *P*>.05). When trying to associate CV and neck pain using chi squared test for forward head and cervical pain, it was clear that neck pain was more prevalent in adolescents with FH than adolescents without FH (29.8% vs 8.4%).

When introducing the sex item, 53% of the girls (n=81) reported NP regularly, contrasting with 19.7% of the boys (n=24). Girls with NP also reported a significantly lower cervical angle than the girls without NP (45.81±5.6 Vs 47.38±4.76º).

Spearman's rho correlation coefficients among the ASES and CV and SH angle are presented in Table 3. None of variables presented a high (r>0.8) and statistically significant correlation other than the expected ASES (right) and ASES (left) (r=0.853).

**Table 3:** Spearman's rho correlations between ASES and the cervical and shoulder angle.

n=275	ASES right	ASES left	Sagittal Head Angle	Cervical Angle	Shoulder Angle
ASES right		0.853* p=0.00	0.031 p=0.592	0.141* p=0.02	-0.001 p=0.0981
ASES left	0.85* p=0.00		0.050 p=0.410	0.141* p=0.004	0.02 p=0.698
Sagittal Head Angle	0.031 p=0.592	0.050 p=0.410		0.07 p=0.245	-0.156 *p=0.010
Cervical Angle	0.141*p=0.02	0.141* p=0.004	0.07 p=0.245		0.057 p=0.293
Shoulder Angle	0.001*p=0.0981	0.02 p=0.698	-0.156 *p=0.010	0.057 p=0.293	

* Correlation is significant at the 0.05 level (2-tailed).

## Discussion

### Reliability study

The present study demonstrated very good reliability for the intra-rater measurements for the HT angle and good reliability for the cervical and shoulder angle in the normal standing posture. With this data, we can suggest that the participants' upper quadrant standing posture did not change significantly over repeated testing. Regarding the inter-rater measurements in the same image for all the variables studied, the very good reliability values are in accordance with the values found by Falla et al.^18^.

### Experimental study

### Descriptive statistics

A large percentage of the subjects revealed some degree of postural abnormality in the cervical and/or shoulder region, with 68% and 58% of the students showing FH and PS, respectively, and 48% of the total sample showing both misalignments.

The incorrect use of heavy backpacks^22^, psychosocial factors such as depression or stress^23^, the lack of ergonomic school furniture^24^, and the extended hours in incorrect postures in school and in front of computers and television^20^may be responsible for this finding.

Specifying the angles studied, we chose HT, CV, and SH angles because they are the most commonly cited in the literature, enabling the comparison of results. These analyses are reliable and help us to characterize a patient's posture in terms of head and shoulder position^8^.

The HT angle measures the alignment of the upper cervical spine^25^. The overall mean HT angle registered (17.2º) is similar to a study by Chansirinukor et al.^15^ with adolescents (13-16 years old) in standing position, which reported a mean HT angle of 16.3º. De Wall et al.^26^ recommended that a suitable HT angle would be 15º above horizontal.

For the CV angle, a smaller angle indicates a more forward head posture^16^. The mean CV angle obtained (47.4º) was similar to the mean reported by van Niekerk et al.^6^ who evaluated 40 adolescents aged 16 to 17 years. In another study with 94 students aged 15 to17 years, Brink et al.^20^ found a smaller CV angle of 39.27º (7.9), which was considered the cause of upper quadrant pain.

The SH angle is an angle that provides a measurement of the shoulder position. The mean SH angle obtained (51º) is the same as the one found by Brink et al.^20^ and very similar to the one found by van Niekerk et al.^6^ (50º). Both studies evaluated adolescents. A smaller angle indicates a PS.

### Effect of postural angles in pain

In an overall view, 105 (38%) participants reported feeling NP regularly. This finding is concurrent with other studies that found a high prevalence of self-reported upper quadrant pain among adolescents^27^, with the shoulder and neck regions becoming more and more cited as the areas of greatest discomfort^28^. Hakala et al.^29^ in a study with adolescents reports NP is common in adolescents, with around one in four reporting NP at least weekly.

This NP can be associated with musculoskeletal disorders, with several studies associating an excessive FH position with NP^8^ ^,^ ^10^ ^,^ ^17^ ^,^ ^30^. For example, Chiu et al.^30^ found that approximately 60% of individuals with NP had FHP. The assumption that greater neck flexion is worse is based on the biomechanical principle relating an increased lever arm (from head center of mass to head/neck and neck/thorax axes of rotation) with increased gross moment. Johnson^31^suggested that prolonged FHP might increase loading to the non-contractile structures and abnormal stress on the posterior cervical structures and cause myofascial pain.

In this study, 68% of the students showed FH, which could predispose then to regular neck pain. Our results confirmed that the adolescents with NP showed a significantly lower CV angle than those without NP (46.5º vs 48.0º). The interdependence between the NP and the CV angles was confirmed with the NP being more prevalent in adolescents with FH than adolescents without FH (29.8% vs 8.4%).

This high prevalence of adolescents with FH and NP can be a reflection of modern Portuguese society, with information technology having a tremendous impact on the life of adolescents through daily use of internet, computers, and console games and with obesity on the rise.

### Effect of sex on the postural angles and pain

Girls showed a lower resting CV angle than boys (46.5º vs 48.4º), which is in accordance with Hakala et al.^29^, who found females had 2-3º more neck flexion than males in a study of standing cervical habitual posture in adolescents. Also in adults, significant sex differences in CV angle have been observed previously, with women having a more forward head position than men^29^. This posture of greater flexion in females can be attributable to psychosocial issues, such as stress, or partly associated with the development of secondary sex characteristics in females.

Contrary to the current study, two studies with small samples reported no sex differences for cervical habitual posture in adolescents and pre-adolescents^2^ ^,^ ^6^. More research is required to clarify the role of sex in cervical posture.

Regarding shoulder posture, we found similar mean values in boys and girls. This is in accordance with Raine and Twomey.^33^, who also reported this similarity in all age groups studied, including the 17-29 age group.

Regarding NP, 52.9% of the girls reported regular NP, contrasting with 19% of the boys. This result is in accordance with previous cross-sectional studies that showed a greater female predisposition to musculoskeletal pain^34^. The reasons for this remain speculative, but we can hypothesize that this result may have been influenced by differences in musculoskeletal systems, such as the fact that girls revealed a significantly lower mean CV angle. Other explanations may be related to differences in behavioral factors, with boys having the tendency to deny pain and girls to overestimate their symptoms at puberty and to have more study-related stress.

## Limitations

The study aimed to minimize errors and bias by recruiting a large sample, setting careful positioning and testing procedures, and blinding the digitization procedure. However it still has some limitations such as the fact that it describes only the alignment of the spine and the shoulder girdle at rest. Therefore the findings cannot be generalized to alignment during functional tasks, especially when the upper limb is moving or loaded.

Another limitation refers to the fact that we have only evaluated the dominant-side. To be more complete, postural alterations could be observed in a non-dominant side as well.

It should also be highlighted that future studies need to characterize the entire spine given the potential influence postures at the lumbar spine have on head position. Also some other variables such as anthropometric variables (e.g. height), degree of thoracic kyphosis or physical activity level must be taken into account.

## Conclusion

The results of the present study showed that the photographic measurement is a reliable tool to assess the standing sagittal posture of the cervical spine and shoulder. It also showed that forward head and protracted shoulder are common postural disorders in adolescents. 68% and 58% of the adolescents revealed anteriorization of the head and protraction of the shoulder, respectively. The subjects with neck pain had a more forward head posture. Sex was also found to have an important effect on posture and neck pain, with girls revealing a lower cervical angle and more neck pain.
